# Primary Pulmonary Non-Hodgkin Lymphoma: A Report of Two Cases and a Review of the Literature Emphasizing the Role of Radiotherapy

**DOI:** 10.7759/cureus.104621

**Published:** 2026-03-03

**Authors:** Veronia Fahmy, Anna Huynh, Alec M Block, James Welsh

**Affiliations:** 1 Radiation Oncology, Loyola University Chicago Stritch School of Medicine, Maywood, USA; 2 College of Medicine, Baylor College of Medicine, Houston, USA; 3 Radiation Oncology, Edward Hines Jr. Veterans Administration (VA) Hospital, Chicago, USA

**Keywords:** diffuse large b-cell lymphoma, extranodal lymphoma, malt lymphoma, marginal zone lymphoma, multimodal therapy, non-hodgkin lung lymphoma, pet/ct, primary pulmonary lymphoma, radiotherapy, rare malignancy

## Abstract

Primary pulmonary non-Hodgkin lymphoma (PPL) is a rare malignancy most commonly presented as indolent marginal zone B-cell lymphoma of mucosa-associated lymphoid tissue (MALT) or bronchus-associated lymphoid tissue (BALT). Aggressive subtypes of PPL, such as diffuse large B-cell lymphoma (DLBCL), occur less frequently. Diagnosis and workup require tissue biopsy, immunophenotyping, and cross-sectional imaging, often supplemented by positron emission tomography (PET)/computed tomography (CT) for staging and treatment planning. Radiation therapy or radiotherapy (RT) is a cornerstone in managing localized, indolent PPL, offering durable local control with minimal toxicity. Modern involved-site RT (ISRT) techniques deliver radiation in conventional fractionation schedules, precisely targeting pulmonary lesions while sparing surrounding lung and mediastinal structures. RT can be used alone for early-stage disease or following systemic therapy for residual or refractory lesions. The cases in this report highlight the importance of histology-driven, individualized treatment planning and the pivotal role of RT in achieving optimal outcomes in this rare malignancy.

## Introduction

Primary pulmonary non-Hodgkin lymphoma (PPL) is a rare and heterogeneous malignancy, accounting for less than 1% of primary pulmonary tumors and extranodal lymphomas [1,2]. The majority of PPLs are indolent marginal zone B-cell lymphomas of mucosa-associated lymphoid tissue (MALT) or bronchus-associated lymphoid tissue (BALT) [1]. However, aggressive subtypes, including diffuse large B-cell lymphoma (DLBCL) and intravascular large B-cell lymphoma, are less frequent and carry poorer outcomes [3].

Radiologically, PPL often mimics more common pulmonary diseases. Recurrent lymphoma presenting as multiple bilateral nodules may resemble metastatic disease, underscoring the need for histopathologic confirmation [4]. The spectrum of clinical presentations ranges from chest wall masses to coexistent tuberculosis, reinforcing the importance of maintaining a broad differential diagnosis [2].

Accurate diagnosis requires integration of tissue biopsy, immunohistochemistry, and cross-sectional imaging, including computed tomography (CT) and fluorine-18 (^18^F) fluorodeoxyglucose (FDG) positron emission tomography (PET)/CT [2]. Taken together, these studies highlight that although rare, PPL demands a high index of suspicion, particularly in patients with atypical radiologic findings, immunosuppression, or unusual extranodal presentations, to ensure timely recognition and appropriate management.

## Case presentation

Case 1

A 78-year-old White male patient with an Eastern Cooperative Oncology Group (ECOG) performance status of 1 was referred for evaluation for definitive radiotherapy (RT) for Ann Arbor stage IE localized pulmonary extranodal marginal zone B-cell lymphoma of MALT type involving the left upper lobe (LUL) [2,5]. The lesion was initially detected in May 2024 on low-dose screening CT, demonstrating a 5.1 × 1.6 × 2.3 cm paramediastinal mass. PET/CT performed in June 2024 showed mild FDG uptake (maximum standardized uptake value (SUVmax) 4.5) without evidence of nodal or distant disease. Bronchoscopy with endobronchial ultrasound-guided biopsy confirmed extranodal marginal zone B-cell lymphoma of MALT type. Immunohistochemistry and flow cytometry demonstrated atypical B-cell proliferation with CD20 and CK AE1/3 positivity, CD5 and CD10 negativity, and CXCR4, MALT1, and MYD88 negativity, consistent with MALT lymphoma [6]. The patient was a current smoker, served in the military during the Vietnam era, and reported no specific occupational exposures.

Following multidisciplinary discussion, surgical resection was not pursued as the patient was not found to be a surgical candidate. The patient received four cycles of rituximab between July and August 2024, resulting in no response on interim PET/CT (SUVmax 4.9). Surveillance was initially elected without consideration for RT. One year later, follow-up PET/CT demonstrated interval progression with enlargement of the primary lesion and new adjacent extranodal involvement confined to the left upper lobe, without evidence of distant disease. Lactate dehydrogenase (LDH) trended as 208 U/L prior to initiating chemotherapy, then rose to 238 U/L in November 2024 and fluctuated back down to 221 U/L on July 29, 2025, throughout this time remaining within normal range (normal value ranges between 140 U/L and 280 U/L) [7]. Pulmonary function testing demonstrated increased residual volume and residual volume/total lung capacity, while the remaining findings were unremarkable. 

**Figure 1:**
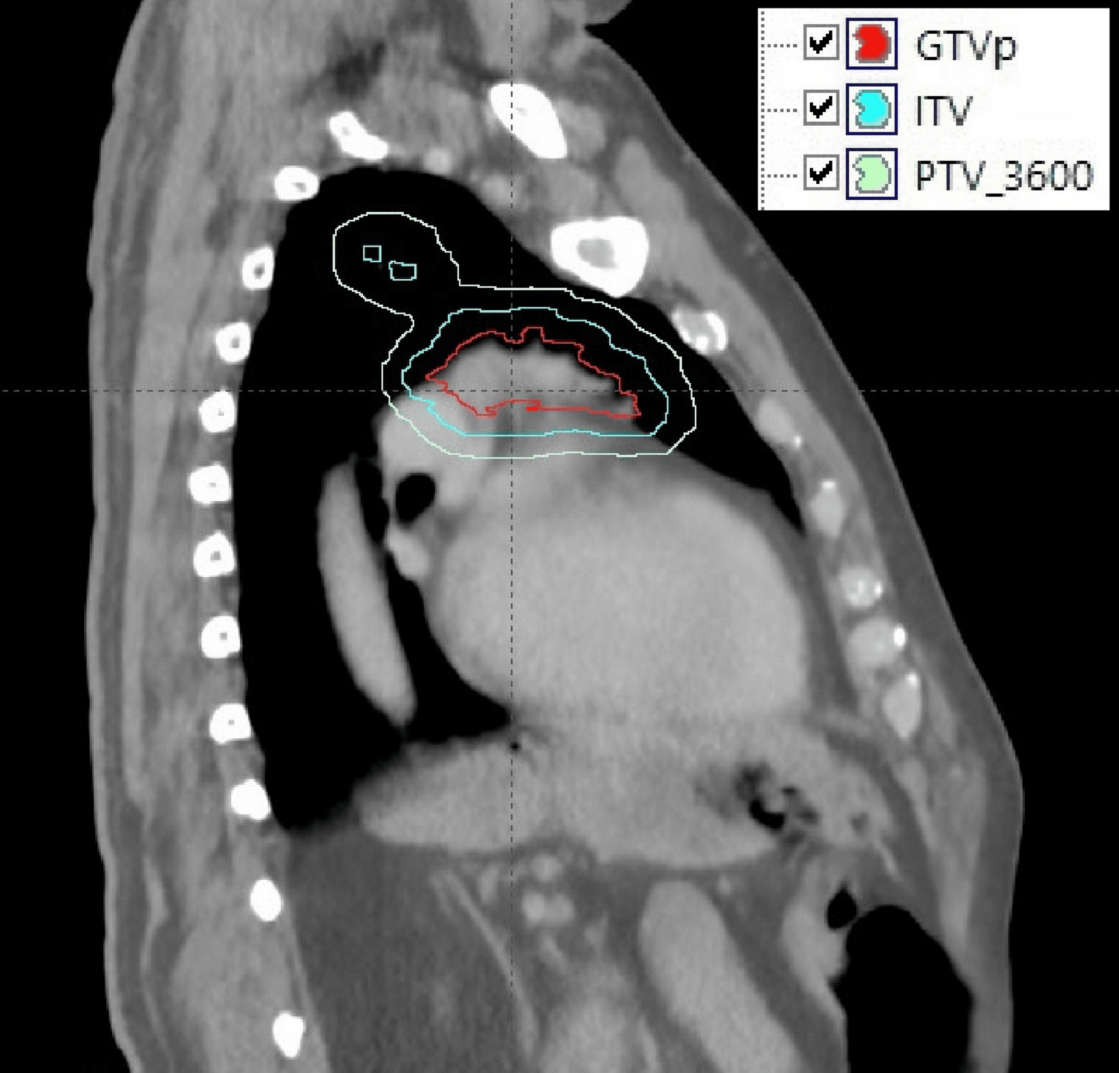
Target volume delineation for involved-site radiotherapy of left upper lobe pulmonary marginal zone lymphoma (Case 1). The GTV contoured on diagnostic imaging is shown. This volume was expanded to an ITV to account for respiratory motion, followed by the addition of a uniform margin to create PTV_3600. The prescribed dose was 36.00 Gy delivered in 18 daily fractions. GTV, gross tumor volume; ITV, internal target volume; PTV_3600, planning target volume receiving 36 Gy; GTVp, gross tumor volume of the primary

The patient subsequently underwent four-dimensional (4D) CT simulation tracking his breathing cycle in preparation for definitive involved-site RT (ISRT) to the left upper lobe without inclusion of mediastinal lymph nodes. As demonstrated in Figure 1, radiation target volume delineation incorporated the gross tumor volume (GTV), utilizing pre-chemotherapy imaging for delineation, and then was expanded by 7 mm to create an internal target volume (ITV) to account for respiratory motion. A uniform margin of 7 mm was applied to generate the planning target volume receiving 36 Gy (PTV_3600). The total prescribed dose was 36 Gy delivered in 18 daily fractions, administered Monday through Friday. 

During RT, the patient experienced minimal toxicity with Common Toxicity Criteria (CTC) scores of 0 for fatigue, skin, lung, and heart, and reported no pain [8]. At three-month follow-up, imaging showed interval improvement: the lesion decreased to 1.25 × 0.27 cm, with SUVmax reduced to 2.5 from 5.5, and no new sites of abnormal uptake were identified. Overall, treatment was well tolerated, and the patient remains under routine surveillance for disease progression or recurrence.

Case 2

A 79-year-old White male patient with an ECOG performance status of 0 was in his usual state of health until May 2025, when he developed progressive congestion and dyspnea. He had a recent diagnosis of congestive heart failure, but was clinically stable from a cardiac standpoint. Pulmonary function testing in June 2025 demonstrated no obstructive or restrictive lung disease. On consultation, he was clinically stable without respiratory distress, with clear lungs to auscultation bilaterally and no use of accessory muscles. The patient was a former smoker, served in the military during the Vietnam era, and reported no specific occupational exposures.

On consultation, the patient was clinically stable without respiratory distress. Pulmonary examination demonstrated clear lungs to auscultation bilaterally, without use of accessory muscles.

CT of the chest performed in June 2025 demonstrated a consolidative lesion in the anterior segment of the left upper lobe measuring 5.6 × 2.0 cm, with an associated regional ground-glass component. The lesion had been previously identified in 2018, measuring 2.6 × 1.4 cm, and was followed on serial CT imaging at that time based on presumed benign etiology. PET/CT demonstrated low-level FDG uptake confined to the left upper lobe lesion, without evidence of additional FDG-avid pulmonary, nodal, or extranodal disease.

Subsequent bronchoscopy with bronchoalveolar lavage demonstrated numerous monotypic B cells. Fine-needle aspiration and biopsy of the lesion revealed a CD5-negative, CD10-negative B-cell lymphoma with plasma cell differentiation. Fluorescence in situ hybridization analysis supported the diagnosis of extranodal marginal zone B-cell lymphoma. Laboratory evaluation, including LDH, was within normal limits [7]. The disease was staged as Ann Arbor stage I [2].

**Figure 2:**
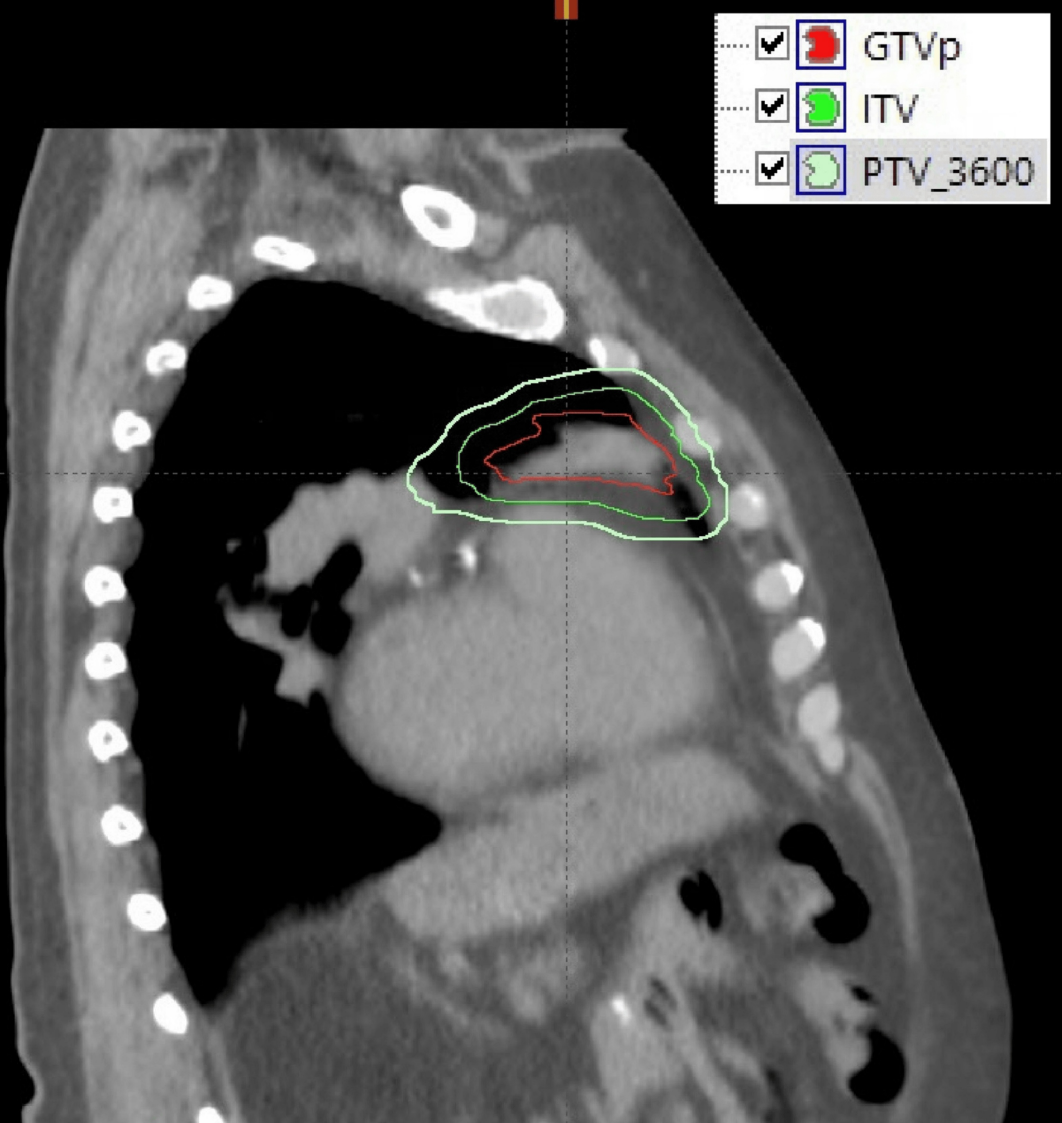
Target volume delineation for left upper lobe pulmonary marginal zone lymphoma (Case 2). The GTV is shown. This volume was expanded to generate the CTV, followed by the addition of a uniform margin to create PTV_3600. GTV, gross tumor volume; ITV, internal target volume; PTV_3600, planning target volume receiving 36 Gy; CTV, clinical target volume; GTVp, gross tumor volume of the primary

Given the localized indolent histology, the patient was treated with definitive ISRT to the left upper lobe without inclusion of regional mediastinal lymph nodes. As demonstrated in Figure 2, radiation target volume delineation incorporated the GTV identified on diagnostic CT and PET imaging, which was expanded by 7 mm to generate the clinical target volume receiving 36 Gy (CTV_3600). A uniform 7 mm margin was applied to create the PTV_3600. The total prescribed dose was 36 Gy delivered in 18 daily fractions, administered Monday through Friday. 

Treatment was well tolerated, with CTC scores of 0 for fatigue, skin, and lung, and the patient reported no pain during therapy. At three months post-treatment, he reported intermittent mild chest wall discomfort, not associated with exertion and resolving within seconds. He denied cough, hemoptysis, dyspnea on exertion, or fatigue. PET/CT demonstrated decreased size and FDG avidity of the treated LUL lesion, now measuring 1.2 × 2.9 cm with SUVmax 1.8, compared to 1.9 × 5.6 cm with SUVmax 3.6 pre-treatment. No additional concerning findings were identified, and the patient remains under routine surveillance.

## Discussion

The management of PPL is highly dependent on histologic subtype and disease extent [2,4]. Indolent MALT/BALT lymphomas often follow a protracted clinical course and may be managed with observation, surgery, immunotherapy, or definitive RT, whereas aggressive subtypes such as DLBCL require systemic anthracycline-based chemotherapy, most commonly cyclophosphamide, doxorubicin, vincristine, and prednisone (CHOP)-based regimens with or without rituximab [9,10]. RT plays a well-established curative role in localized, indolent pulmonary lymphoma, with doses of 30-40 Gy delivered in conventional fractionation, achieving excellent local control and long-term disease stabilization in early-stage MALT lymphoma [9].

Published series describing RT dose, fractionation, and timing in PPL are summarized in Table 1. Modern techniques typically use involved-site fields with doses ranging from 24-36 Gy, depending on histology, while minimizing exposure to healthy lung tissue and nearby organs [11]. Pulmonary presentations of lymphoma are often associated with autoimmune diseases such as Sjögren’s syndrome, rheumatoid arthritis, or systemic lupus erythematosus, as well as chronic inflammation and immunosuppressive therapy, including methotrexate exposure [12]. Low-grade marginal zone lymphoma is frequently localized at diagnosis and responds well to monochemotherapy with chlorambucil, showing favorable long-term survival even in patients with autoimmune comorbidities [13,14]. In contrast, the role of RT is more limited in disseminated disease or aggressive histologies, where systemic therapy remains the cornerstone of management [10].

**Table 1:** Clinicopathologic characteristics and treatment of pulmonary non-Hodgkin lymphoma RT, radiotherapy; MALT, mucosa-associated lymphoid tissue; BALT, bronchus-associated lymphoid tissue

Study	Histologic Subtype	Median Age (years)	Primary Site	Treatment Approach	Key Outcomes
Zinzani et al. (2008) [10]	B-cell non-Hodgkin lymphoma	Not mentioned	Pulmonary ± mediastina	Chemotherapy + RT	Durable disease control
Milosević et al. (2011) [13]	MALT lymphoma	~60	Lung	Surgery, rituximab ± RT	Excellent overall survival at four-year follow-up
Kocatürk et al. (2012) [15]	MALT lymphoma	Not mentioned	Lung	Surgery ± RT	Excellent local control
Savari et al. (2018) [16]	MALT (neuroendocrine variant)	Not mentioned	Pulmonary	Chemotherapy + rituximab ± RT	Favorable prognosis
Zhang et al. (2019) [17]	Predominantly MALT	54	Pulmonary	Surgery, rituximab, chemotherapy ± RT	High overall and progression-free survival
Hu et al. (2022) [2]	Predominantly MALT	57	Pulmonary	Rituximab-based therapy ± RT	Improved local control with RT
Wu et al. (2022) [1]	MALT lymphoma	55	Pulmonary	Surgery, rituximab ± RT	Excellent long-term control

The present cases illustrate the clinical heterogeneity of PPL and the importance of histology-driven treatment. In the first case, systemic therapy achieved only temporary disease stabilization, with definitive RT providing local control at progression. In the second case, upfront RT was selected for localized indolent disease, avoiding systemic therapy while achieving disease control. These cases underscore the value of PET/CT staging, tissue confirmation, and individualized treatment planning. 

Further, extranodal marginal zone lymphoma can coexist with neuroendocrine differentiation, emphasizing the importance of detailed immunohistochemical evaluation for accurate diagnosis and characterization [16]. Rare aggressive variants such as intravascular large B-cell lymphoma further emphasize the need for precise histopathologic and molecular characterization [3]. Additionally, high-grade transformation of indolent MALT lymphoma to DLBCL has been reported, highlighting the necessity of long-term surveillance [9]. Most patients present with nonspecific respiratory symptoms, the majority have indolent histology such as MALT lymphoma, and outcomes are generally favorable with histology-directed therapy [17].

Primary lung or pleural lymphoma is extremely rare, accounting for only 0.7% of B-cell lymphomas, while secondary involvement occurs more frequently, predominantly in DLBCL [18]. Lung involvement often reflects systemic disease rather than a primary pulmonary origin. Although primary chest wall lymphoma is uncommon, surgical excision followed by adjuvant chemotherapy has provided excellent long-term disease control in localized cases, highlighting the benefit of combined modality therapy in selected extranodal presentations [19]. Primary pulmonary DLBCL is exceptionally rare and often presents with nonspecific respiratory symptoms, making timely diagnosis challenging and emphasizing the need for careful histopathological evaluation to guide treatment [20]. Pulmonary involvement in non-Hodgkin lymphoma is uncommon, but diffuse ground-glass opacities on CT should prompt consideration of DLBCL, as they may reflect atypical infiltration of the alveolar septa and bronchiolar walls rather than nodular disease [21]. ^18^F-FDG PET/CT plays an important role in the initial staging and response assessment of PPL. While FDG uptake in indolent MALT lymphoma is often lower and more variable than in aggressive subtypes such as DLBCL, most cases remain FDG-avid and detectable with modern PET/CT techniques, supporting its use in both diagnosis and treatment planning [22].

Overall, PPL generally carries a more favorable prognosis than other primary lung malignancies, with marginal zone MALT lymphoma representing the most common subtype [23]. For patients with localized indolent PPL, RT is the preferred definitive treatment, achieving excellent local control with minimal toxicity. For aggressive histologies or disseminated disease, systemic therapy remains the cornerstone, with RT serving primarily as a consolidative or palliative modality [24].

## Conclusions

RT remains a cornerstone in the management of localized, indolent PPL, particularly MALT/BALT subtypes. Historically, treatment approaches for extranodal lymphomas often involved larger radiation fields and higher doses; however, advances in imaging, staging, and radiation planning have enabled a shift toward involved-site techniques that prioritize precise localization while limiting exposure to adjacent normal structures. Contemporary RT strategies achieve excellent local control with reduced toxicity compared to earlier approaches.

While systemic therapy remains the mainstay for aggressive or disseminated disease, selected patients with localized progression may benefit from targeted RT. These findings underscore the importance of individualized, histology-driven treatment planning and long-term surveillance to optimize outcomes.

## References

[1] Wu T, Huang Y, Wang Z, Cao H, Ding Q, Deng Z (2022). Pulmonary MALT lymphoma: imaging findings in 18 cases and the associated pathological correlations. Am J Med Sci.

[2] Hu M, Gu W, Chen S, Mei J, Wang W (2022). Clinical analysis of 50 cases of primary pulmonary lymphoma: a retrospective study and literature review. Technol Cancer Res Treat.

[3] Yu H, Chen G, Zhang R, Jin X (2012). Primary intravascular large B-cell lymphoma of lung: a report of one case and review. Diagn Pathol.

[4] Yao D, Zhang L, Wu PL, Gu XL, Chen YF, Wang LX, Huang XY (2018). Clinical and misdiagnosed analysis of primary pulmonary lymphoma: a retrospective study. BMC Cancer.

[5] Oken MM, Creech RH, Tormey DC, Horton J, Davis TE, McFadden ET, Carbone PP (1982). Toxicity and response criteria of the Eastern Cooperative Oncology Group. Am J Clin Oncol.

[6] Bagratuni T, Ntanasis-Stathopoulos I, Gavriatopoulou M (2018). Detection of MYD88 and CXCR4 mutations in cell-free DNA of patients with IgM monoclonal gammopathies. Leukemia.

[7] Farhana A, Lappin SL (2023). Biochemistry, lactate dehydrogenase. StatPearls [Internet].

[8] (1999). Common Toxicity Criteria (CTC), version 2.0. https://dctd.cancer.gov/research/ctep-trials/for-sites/adverse-events/ctc-v2.pdf.

[9] Yahalom J, Illidge T, Specht L, Hoppe RT, Li YX, Tsang R, Wirth A (2015). Modern radiation therapy for extranodal lymphomas: field and dose guidelines from the International Lymphoma Radiation Oncology Group. Int J Radiat Oncol Biol Phys.

[10] Zinzani PL, Martelli M, Poletti V (2008). Practice guidelines for the management of extranodal non-Hodgkin's lymphomas of adult non-immunodeficient patients. Part I: primary lung and mediastinal lymphomas. A project of the Italian Society of Hematology, the Italian Society of Experimental Hematology and the Italian Group for Bone Marrow Transplantation. Haematologica.

[11] Cahan B, Chen YJ (2015). Modern radiation therapy for extranodal lymphomas: field and dose guidelines from the International Lymphoma Radiation Oncology Group: in regard to Yahalom et al. Int J Radiat Oncol Biol Phys.

[12] Yachoui R, Leon C, Sitwala K, Kreidy M (2017). Pulmonary MALT lymphoma in patients with Sjögren’s syndrome. Clin Med Res.

[13] Milosević V, Bogdanović A, Janković S, Jovanović MP, Mihaljević B (2011). Extranodal marginal zone non-Hodgkin's lymphoma of the lung: a ten-year experience. (Article in Serbian). Vojnosanit Pregl.

[14] Cheah CY, Zucca E, Rossi D, Habermann TM (2022). Marginal zone lymphoma: present status and future perspectives. Haematologica.

[15] Kocatürk Cİ, Seyhan EC, Günlüoğlu MZ, Urer N, Kaynak K, Dinçer Sİ, Bedirhan MA (2012). Primary pulmonary non-Hodgkin's lymphoma: ten cases with a review of the literature. Tuberk Toraks.

[16] Savari O, Hastings H, Rayes R, Tomashefski JF Jr (2018). Neuroendocrine neoplasia in extranodal marginal zone lymphoma of mucosa-associated lymphoid tissue (MALT lymphoma) of the lung: a case report and immunohistochemistry analysis of eight pulmonary MALT lymphomas. Int J Surg Pathol.

[17] Zhang XY, Gu DM, Guo JJ, Su QQ, Chen YB (2019). Primary pulmonary lymphoma: a retrospective analysis of 27 cases in a single tertiary hospital. Am J Med Sci.

[18] Mian M, Wasle I, Gritsch S, Willenbacher W, Fiegl M (2015). B cell lymphoma with lung involvement: what is it about?. Acta Haematol.

[19] Hsu PK, Hsu HS, Li AF, Wang LS, Huang BS, Huang MH, Hsu WH (2006). Non-Hodgkin's lymphoma presenting as a large chest wall mass. Ann Thorac Surg.

[20] Zhu Z, Liu W, Mamlouk O, O'Donnell JE, Sen D, Avezbakiyev B (2017). Primary pulmonary diffuse large B cell non-Hodgkin’s lymphoma: a case report and literature review. Am J Case Rep.

[21] Tokuyasu H, Harada T, Watanabe E (2009). Non-Hodgkin's lymphoma accompanied by pulmonary involvement with diffuse ground-glass opacity on chest CT: a report of 2 cases. Intern Med.

[22] Peng Y, Qi W, Luo Z (2022). Role of 18F-FDG PET/CT in patients affected by pulmonary primary lymphoma. Front Oncol.

[23] Wannesson L, Cavalli F, Zucca E (2005). Primary pulmonary lymphoma: current status. Clin Lymphoma Myeloma.

[24] Ziadé N, Khayat G, Sader-Ghorra C, Abadjian G (2005). Primary pulmonary lymphoma. A case report and review of the literature. (Article in French). J Med Liban.

